# Discovery of Colossal Breathing-Caloric Effect under Low Applied Pressure in the Hybrid Organic–Inorganic MIL-53(Al) Material

**DOI:** 10.1021/acs.chemmater.2c00137

**Published:** 2022-03-30

**Authors:** Javier García-Ben, Jorge López-Beceiro, Ramon Artiaga, Jorge Salgado-Beceiro, Ignacio Delgado-Ferreiro, Yury V. Kolen’ko, Socorro Castro-García, María Antonia Señarís-Rodríguez, Manuel Sánchez-Andújar, Juan Manuel Bermúdez-García

**Affiliations:** † Quimolmat, Centro de Investigacións Científicas Avanzadas (CICA), Universidade da Coruña,, Rúa as Carballeiras, 15071 A Coruña, Spain; ‡ Quimolmat, Departamento de Química, Facultade de Ciencias, Universidade da Coruña, Campus da Zapateira, 15008 A Coruña, Spain; § Escuela Politécnica de Ingeniería de Ferrol, Universidade da Coruña, Campus Industrial de Ferrol, 15403 Ferrol, A Coruña, Spain; ⊥ International Iberian Nanotechnology Laboratory (INL), Avenida Mestre José Veiga, 4715-330 Braga, Portugal

## Abstract

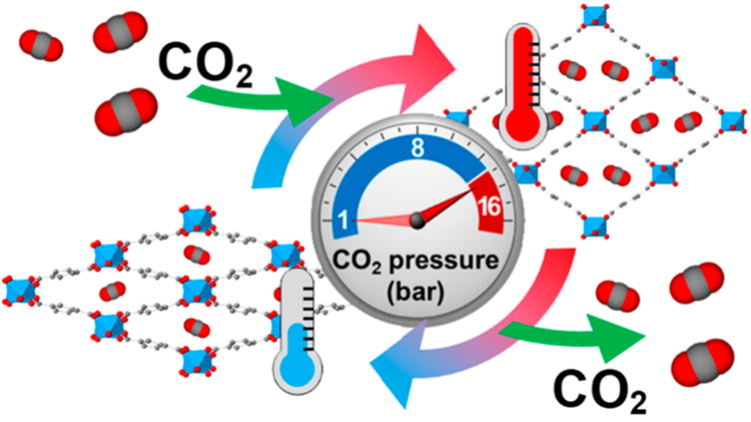

In this work, “breathing-caloric” effect is introduced as a new term to define very large thermal changes that arise from the combination of structural changes and gas adsorption processes occurring during breathing transitions. In regard to cooling and heating applications, this innovative caloric effect appears under very low working pressures and in a wide operating temperature range. This phenomenon, whose origin is analyzed in depth, is observed and reported here for the first time in the porous hybrid organic–inorganic MIL-53(Al) material. This MOF compound exhibits colossal thermal changes of Δ*S* ∼ 311 J K^–1^ kg^–1^ and Δ*H* ∼ 93 kJ kg^–1^ at room temperature (298 K) and under only 16 bar, pressure which is similar to that of common gas refrigerants at the same operating temperature (for instance, *p*(CO_2_) ∼ 64 bar and *p*(R134a) ∼ 6 bar) and noticeably lower than *p* > 1000 bar of most solid barocaloric materials. Furthermore, MIL-53(Al) can operate in a very wide temperature range from 333 K down to 254 K, matching the operating requirements of most HVAC systems. Therefore, these findings offer new eco-friendly alternatives to the current refrigeration systems that can be easily adapted to existing technologies and open the door to the innovation of future cooling systems yet to be developed.

## 1 Introduction

As Professor Goodenough used to emphasize to his students, energy is one of the crucial challenges facing mankind, and solid-state chemists have the power of providing materials for a more sustainable world. On this basis we dedicate this work to him, on the occasion of his 100th birthday.

Around 20% of the world’s electricity consumption is devoted to refrigeration technologies, such as fridges, freezers, HVAC (heating, ventilation, and air conditioning) systems, etc.^1,2^ Moreover, demand on this sector is expected to markedly grow in the coming decades due to global warming.^2,3^ Actually, the current COVID-19 pandemic has put under the spotlight the great importance of refrigeration. For instance, one of the most pressing challenges is still the refrigeration of COVID-19 vaccines, a major barrier that limits worldwide distribution and access.^4^

Nowadays, most refrigeration technologies are still based on compression/expansion cycles of volatile gases, first exploited over 180 years ago. This technology, although mature and well-established, still operates well below its maximum theoretical thermodynamic efficiency. In turn, refrigeration systems account for 7% of the global greenhouse gas emissions,^2,3^ where 5% is due to indirect emissions from the inefficient energy consumption, and 2% is due to direct emissions of gas refrigerants, mainly fluorinated hydrocarbons (F-gases) with global warming potential (GWP) thousands of times larger than that of CO_2_. Therefore, the Kigali Agreement and F-gas regulation (EU regulation no. 517/2014) urge phase-out of 80% of these F-gas refrigerants by 2030. In this pressing scenario, and looking for alternatives, solid-state materials that can present pressure-induced phase transitions are arising as a promising alternative to refrigerant gases. These solid materials, known as barocaloric compounds, can exhibit large thermal changes (isothermal entropy changes Δ*S* or adiabatic temperature changes Δ*T*) when undergoing a solid to solid phase transition induced by the application and removal of isostatic pressure, in a similar way to refrigerant gases.^5−11^ In general, barocaloric materials can offer many advantages over gas refrigerants: they cannot escape to the atmosphere, they are easier to recover and reuse in case of system breakage, they can be transported in nonpressurized vessels, and they can lead toward more compact systems, among others.

Nevertheless, it remains a major challenge finding materials that comply with all three main requirements for commercial refrigeration: (i) very large thermal changes, (ii) low operating pressures (similar to the current cooling systems), and (iii) wide operating temperature range near room temperature. For example, recently discovered barocaloric organic plastic crystals exhibit unprecedentedly large thermal changes that can be comparable to those observed in commercial refrigerants.^12−14^ However, they require very high operating pressures between 1000 and 2500 bar,^12−14^ which are still far from the working pressure of conventional systems (*p* < 150 bar).

On the other hand, our group recently reported hybrid organic–inorganic perovskites as the first barocaloric materials working under 70 bar,^15−17^ a pressure much closer to commercial needs. However, these compounds exhibit noticeably smaller entropy changes than organic plastic crystals. Moreover, in both families of barocaloric materials the operating temperature range (temperature span) is still limited and should be improved.

In parallel, and looking for alternatives to refrigeration based on compression/decompression, porous hybrid materials, specifically MOFs (metal–organic frameworks), have been explored for adsorption-driven heat pump technologies, taking advantage of their remarkable adsorption properties. In this case, and differently from the aforementioned barocalorics, the cooling effect is induced by the vaporization enthalpy upon ambient temperature change. Here the enthalpy change is associated with the thermally induced release of an adsorbate (mainly water but also other small molecules such as methanol and/or CO_2_) initially present in the cavities of the porous MOFs.^18−22^

Adsorption refrigeration shows remarkable advantages over vapor-compression and barocaloric cooling, such as the possibility of using residual heat as the driving stimuli for operating (instead of pressure) or avoiding the use of moving parts (such as the compressor) and their noise/vibrations, among others.^23^ However, it also has important drawbacks, such as relatively low efficiency and complex technological designs that require high vacuum.^23^

Remarkably enough, and according to a very recent publication, porous MOFs with breathing transitions could also be of interest for refrigeration under pressure.^10^ Breathing transitions are first-order solid to solid phase transitions that occur between two crystalline phases of MOFs with differences in their pore size (the lower volume narrow-pore phase, np, and the larger volume large-pore phase, lp). Such transitions take place upon uptake/release of gas, depending on the specific MOF.^24,25^ This behavior resembles the lungs’ breathing mechanism, which gives rise to the aforementioned name. In view of the large volume changes which are associated with this type of phase transition, such materials could exhibit potentially interesting pressure-induced thermal changes similar to those of solid-state barocaloric refrigerants as indicated by D. Boldrin.^10^

For the present work and to experimentally study such a possibility, we have reviewed the literature on breathing-MOFs, identifying a material with a breathing transition near room temperature under pressures below 10 bar^26−29^ and which could be an ideal potential candidate to exhibit very large pressure-induced caloric effects for commercial refrigeration applications: the MIL-53(Al) compound.

**Figure 1:**
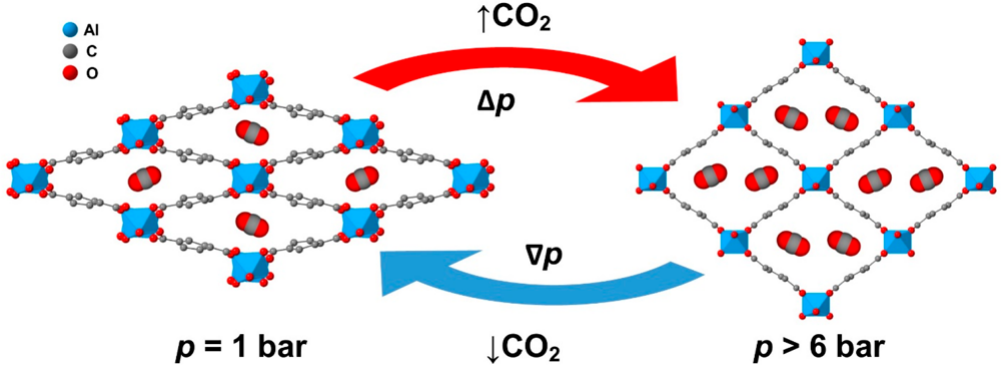
Representation of the narrow-pore np-phase (left) and large-pore lp-phase (right) of MIL-53(Al) viewed along the axis of the unidimensional channels under compression and decompression with CO_2_.^26^ Note: CO_2_ molecules have been randomly allocated for visualization purposes.

From the chemical point of view, this compound exhibits the molecular formula Al(OH)[BDC]·[G], where BDC = 1,4-benzenedicarboxylate [C_6_H_4_(CO_2_)_2_]^2–^ anions; G = different guest molecules which can be adsorbed in the material’s cavities and can range from small gas molecules (H_2_, CO_2_) to even large dyes (methylene blue).^30,31^ Meanwhile, from the structural point of view, the MIL-53(Al) framework topology is formed by unidimensional chains of corner-sharing Al(BDC)_4_(OH)_2_ octahedra linked by BDC ligands, which results in linear lozenge-shaped channels large enough to accommodate the guest molecules (Figure 1).

For our study and to induce the breathing transition, we have selected CO_2_ as the pressurized gas (see Figure 1), which not only induces structural changes from the np- to the lp-phase but also chemically interacts with the framework, creating/breaking hydrogen bonds upon adsorption/desorption.^28^

In this context, it should also be noted that CO_2_ is a common refrigerant whose use is spreading within the commercial refrigeration sector due to its low global warming potential (GWP). Moreover, although CO_2_ is undoubtedly one of the most well-known greenhouse gases, for the application of refrigeration it can be extracted from residual gases in industrial processes.^32^ Additionally, the capture systems for extracting CO_2_ from the atmosphere are growing increasingly cost-effective.^33^ In fact, one of the main strategies to keep CO_2_ out of the atmosphere, besides minimizing its production, is to capture and reuse it in long-term industrial applications.^32^ Therefore, the use of CO_2_ in refrigeration does not contribute to global warming but rather the opposite: its use in refrigeration would be a carbon neutral process. Moreover, this gas is chemically stable, nontoxic, nonflammable, widely available, and low cost.^34−37^

In the present experimental work, we investigate the thermodynamic response of the MIL-53(Al) breathing transition under different external stimuli (temperature and pressure), and we analyze its potential for refrigeration applications. As we will show, the obtained results reveal very large caloric effects induced by pressurization/depressurization cycles of CO_2_, which are comparable in magnitude, and also in operating pressure and temperature, with commercial gases. These findings provide a fundamental basis for an innovative caloric refrigeration mechanism (breathing-caloric effect, as will be properly defined below) and also open the door for future practical implementation of MOFs in new eco-friendly refrigeration technologies.

## 2 Experimental Section

### 2.1 Reagents

Commercially available analytical grade Al(NO_3_)_3_·9H_2_O (>98% ACS reagent) and terephthalic acid (98% Sigma-Aldrich) were used as purchased without any further purification.

### 2.2 Synthesis of MIL-53(Al)

MIL-53(Al) was synthesized following the literature conditions.^38^ Specifically, the synthesis was carried out in a stainless-steel hydrothermal reactor containing a 50 mL PTFE vessel. A deionized-water solution of 1.99 g (5.3 mmol) of Al(NO_3_)_3_·9H_2_O and 1.38 g (8.3 mmol) of terephthalic acid was placed in the PTFE vessel. The hermetically closed reactor was heated and maintained at 493 K during 4 days. After this time, a white solid precipitate was obtained. This precipitate was filtered, washed with deionized water, and heated at 603 K for 3 days to remove the solvent–guest molecules from the material’s cavities, following the reported procedure.^38^

### 2.3 Powder X-ray Diffraction

The obtained material was characterized by powder X-ray diffraction (PXRD) using a Siemens D-5000 diffractometer with Cu Kα radiation at room temperature. The obtained patterns were compared with those simulated from reported single-crystal XRD.^38^

### 2.4 Fourier Transform Infrared Spectroscopy

The infrared spectra of the solid product were analyzed on a powdered sample of MIL-53(Al) on a Thermo Scientific Nicolet iS10 FT-IR spectrometer, in the range of 500 to 4000 cm^–1^.

### 2.5 Transmission Electron Microscopy

The size and morphology of the samples was studied by transmission electron microscopy (TEM) using a JEOL 1010 microscope operating at 100 kV. For that purpose, the samples were suspended in 2-propanol and deposited onto copper grids.

### 2.6 BET Characterization

Nitrogen adsorption/desorption isotherms were carried out using an ASAP 2020 Micromeritics equipment. For the degasification, the sample was dried at 423 K during 24 h. The nitrogen adsorption/desorption isotherm was measured under nitrogen atmosphere at 77 K.

### 2.7 Calorimetry Studies

Variable-temperature (VT) and variable-pressure (VP) differential scanning calorimetry (DSC) tests were performed in a TA Instruments pressure-cell mounted on a Q2000 MDSC (modulated differential scanning calorimeter). In a homemade upgrade of the calorimeter (see Figure S1 of Supporting Information, SI), the gas-pressure in the input line is controlled by a Bronkhorst EL-PRESS P-802CV automatic regulator. Meanwhile, a Bronkhorst EL-FLOW Select F-201CV flux controller is placed at the output line. Variable-temperature (VT) measurements were performed only upon heating at 10 K min^–1^ between 310 and 410 K at different constant pressures (1–5 bar). The absence of a cold source coupled with the pressure-cell hindered a controlled cooling ramp and the quantitatively calorimetric analysis on cooling. Variable-pressure (VP) measurements were performed under pressurization/depressurization ramps of CO_2_ at different rates (0.6–1.6 bar min^–1^) and different constant temperatures (298–333 K) and maintaining a constant flux of 50 mL min^–1^. All the experiments were performed on a ∼5 mg sample, previously dried inside the equipment under CO_2_ at 473 K for 10 min. The calorimeter coupled with the pressure-cell was calibrated according to the manufacturer recommendations. The baseline slope and offset of VT-DSC curves were calibrated by heating the empty cell throughout the entire temperature range to be used in the experiments. The small effect of pressure on the temperature and enthalpy calibration was verified with indium melting in the experimental range. The variation observed was, in all cases, less than 0.2 K and less than 1% of enthalpy, see Figure S2 of SI. In the case of VP-DSC experiments, the baseline was corrected by subtracting the calorimetric curves obtained by empty pans under CO_2_ pressurization and depressurization in the experimental range. It should be also noted that before performing any DSC measurement on MIL-53(Al), we removed any possible adsorbed moisture water by drying the sample at 423 K in CO_2_ atmosphere at ambient pressure (∼1 bar).

### 2.8 Thermal Analysis

Thermogravimetric analysis (TGA) was carried out using TGA-DTA thermal analysis SDT2960 equipment. The experiment was set up with 5 mg of the prepared powder sample with a ramp of 10 K min^–1^ from 300 to 1200 K, using an alumina crucible, and under a 100 mL min^–1^ flow of dry nitrogen.

## 3 Results and Discussion

### 3.1 Basic Characterization of the MIL-53 Sample

We have confirmed the purity of the prepared material by powder X-ray diffraction and FT-IR spectroscopy (see Experimental Section and Figures S3 and S4 of SI). The slight discrepancy in the intensity of some peaks of the experimental PXRD pattern compared to that simulated from reported single crystal XRD^38^ can be attributed to preferred orientations in the sample favored by the shape of the particles, rhomboid platelets with a size of 1.1 μm and a dispersion of ±0.4 μm according to TEM (see Figure S5 of SI).

In addition, according to TGA data (see Figure S6 of SI), the obtained sample captures water from the environment (∼8% in weight), that is eliminated when heating it above 373 K. As for the porosity of the obtained material, according to BET analysis, it shows a surface area of ∼1400 m^2^ g^–1^ (see Figure S7 of SI), which is in good agreement with the literature considering the reported porosity dispersion depending on the sample (see comparison of our experimental data with those reported in Figure S7 of SI).^39,40^

### 3.2 Thermodynamic Response of the Breathing Transition under Different External Stimuli: Temperature and Pressure

To investigate the thermodynamic response of the MIL-53(Al) breathing transition and explore its potential for refrigeration, we have studied the response of this material under different external stimuli (such as temperature and applied isostatic pressure) under CO_2_ atmosphere by DSC.

In all those experiments, the starting conditions were room temperature and ambient pressure (under CO_2_ atmosphere), under which the MIL-53(Al) material is in the np-phase with several CO_2_ molecules inside the cavities.^26,27^ Therefore, the applied external stimuli (temperature and/or pressure) will provoke a reversible breathing transition between np- and lp-phase (see Figure 1).

#### 3.2.1 Temperature Influence under Different Isobaric Conditions

**Figure 2:**
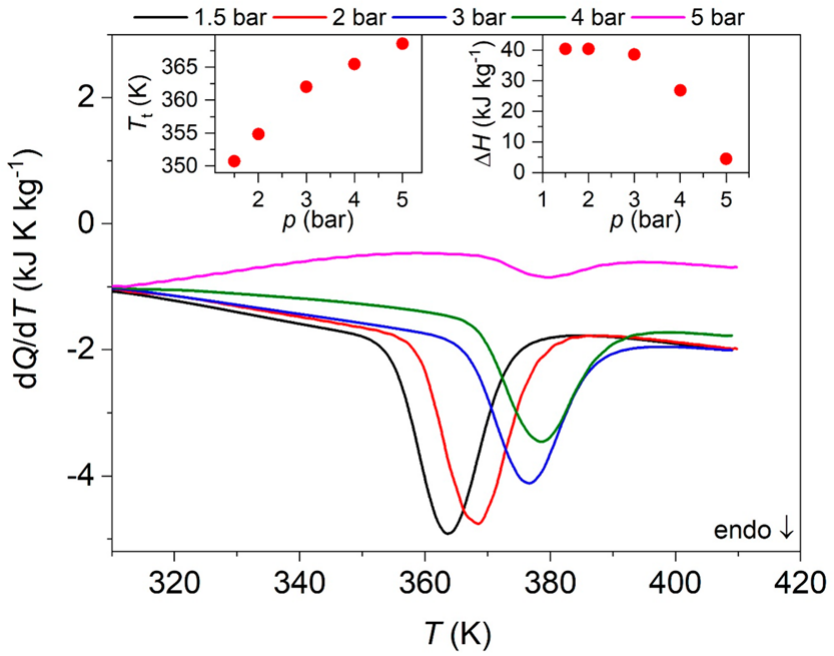
VT-DSC curve on heating ramps (10 K min^–1^) under different CO_2_ isobaric conditions (*p* = [1.5–5] bar). Left inset: pressure dependence of the transition temperature. Right inset: pressure dependence of the latent heat.

For studying the influence of temperature on the breathing transition, we performed variable-temperature (VT) DSC studies under different CO_2_ isobaric conditions (*p* = [1.5–5] bar). Figure 2 shows the obtained DSC curves, which reveal the endothermic peak associated with the first-order transition from the np-phase to the lp-phase at different pressures. Very interestingly, and as can be seen there, the transition temperature markedly shifts toward higher temperatures as the CO_2_ pressure increases. Actually, such displacement is as large as d*T*_t_/d*p* ∼ 7.5 K bar^–1^ for *p* < 3 bar and 3.3 K bar^–1^ for *p* > 3 bar (see Figure 2 left inset), a value that is 2 orders of magnitude larger than in any reported barocaloric material.^6^

On the other hand, the latent heat associated with the breathing transition is seen to largely decrease for CO_2_ pressures above 3 bar, almost disappearing at 5 bar (see Figure 2 right inset). It is worth noting that, according to the literature, such latent heat consists of two major contributions, one associated with the CO_2_ adsorption and another with lattice effects.^26−29^ Both effects occur at the same time; therefore, it is challenging to identify the thermal changes of each individual process.

In this context, molecular dynamics simulations indicate that the lattice effect from np-phase to lp-phase is endothermic with an enthalpy change value of Δ*H* ∼ 43 kJ kg^–1^.^28^ Therefore, for *p* < 3 bar, we can assume that, the main contribution of the thermally induced phase transition observed here corresponds to the structural transition from np-phase to lp-phase alone (lattice effect). To rationalize the results observed at higher pressures, where the value of Δ*H* decreases sharply, we suggest that CO_2_ adsorption (exothermic process) will be favored in this interval.

**Figure 3:**
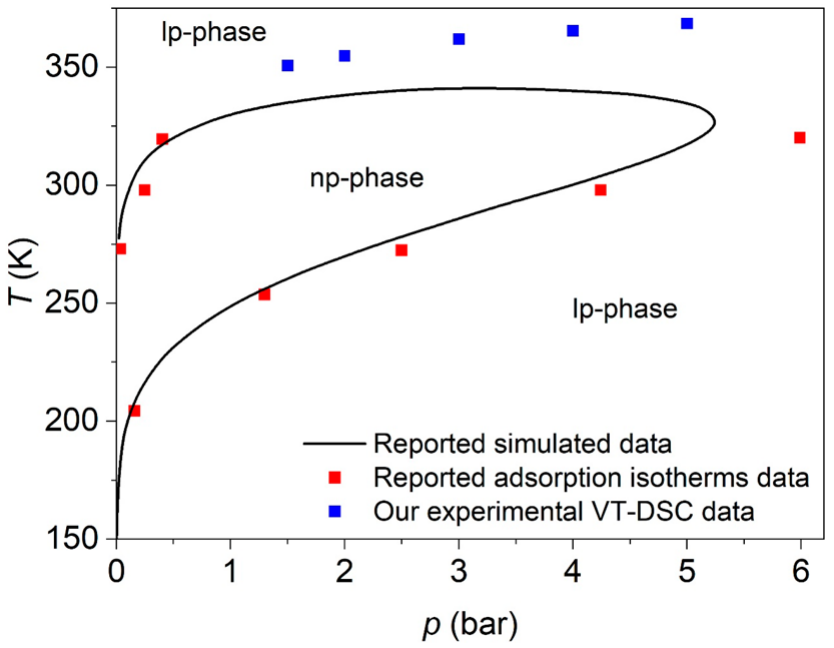
Pressure–temperature phase diagram of MIL-53(Al) under CO_2_ atmosphere, where the solid black line represents the reported simulated profile calculated by adsorption isotherms data, the red points indicate the reported experimental points obtained by adsorption isotherms, both taken from ref (26), and the blue points represent the experimental data obtained by VT-DSC in the present work.

In any case, the transition temperature is undoubtedly related to the structural transition between the np-phase and the lp-phase. Actually, these data help to complete the *p*–*T* phase diagram previously reported in the literature^26^ as shown in Figure 3, where the simulated profile previously calculated from adsorption isotherms data, together with a few previous experimental points in the low temperature region, is now completed in the high temperature range by the data obtained here (blue points).

#### 3.2.2 Pressure Influence under Different Isothermal Conditions

For studying the influence of pressure on the breathing transition, we have carried out variable-pressure (VP) DSC analysis under different isothermal conditions. These studies are very useful to better characterize the potential refrigeration capability of a material, given that traditional cooling devices operate under pressurization/depressurization cycles. However, due to the complexity of such experiments and the specific equipment required they still remain very scarce in the literature.

**Figure 4:**
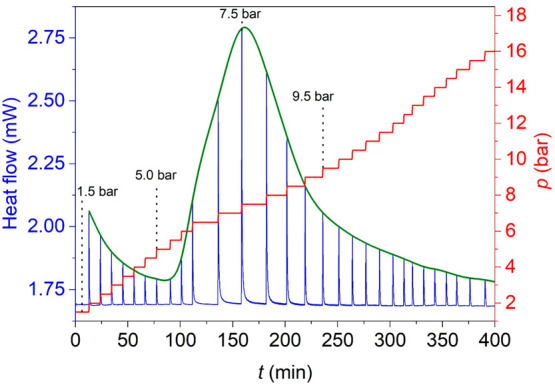
Pressure and heat flow signals obtained during CO_2_ adsorption on MIL-53(Al) at 300 K using a point-by-point procedure of gas introduction.

For this purpose, we first perform a point-by-point pressure adsorption calorimetric analysis,^41^ increasing the CO_2_ pressure in steps of 0.5 bar from 1.5 to 16 bar under isothermal conditions at ∼300 K and registering the heat flow (see Figure 4). As shown there, each step of pressure increase generates a sharp, lambda shape peak with a small narrow width of ∼2 min at the base, where the enthalpy change is directly related to the area under the peaks.

As for the origin of the changes in enthalpy upon compression, in view of their exothermic nature, in all cases we can conclude that CO_2_ adsorption is the prevalent contribution in the observed behavior. On this basis and taking into account the variation of Δ*H* upon compression for the different pressures values, we can rationalize the observed behavior as follows: From ambient pressure up to 5.5 bar, Δ*H* decreases upon pressurization (from 3.6 kJ kg^–1^ at 1.5 bar down to 1.4 kJ kg^–1^ at 5.5 bar) due to the progressive saturation of the MIL-53(Al) pores in the np-phase. Second, when increasing the CO_2_ pressure from 5.5 bar up to 7.5 bar, the structural transition from np-phase to the lp-phase takes place (breathing transition). This provokes an increase of the Δ*H* value (with a maximum of Δ*H* ∼ 16.5 kJ kg^–1^ at 7.5 bar), which can be related to the enhanced adsorption capacity of the induced lp-phase. Third, from 7.5 up to 16 bar, the value of Δ*H* keeps decreasing due to a further saturation of the lp-phase pores. It should be noted that the experimental data reported here are fully in agreement with the experimental and theoretical data reported in the literature.^26,27,29^

From these point-by-point calorimetric data at 300 K (Figure 4), we can estimate the total enthalpy change of the complete pressurization process (from 1.5 to 16 bar) as the sum of the enthalpy change of each one of these steps. We obtain a value of Δ*H*_(1.5→16 bar)_ ∼ 106.8 kJ kg^–1^, and this would be the maximum Δ*H* value at *p* ∼ 16 bar, allowing the material to reach the thermodynamic equilibrium over a time of 400 min.

**Figure 5:**
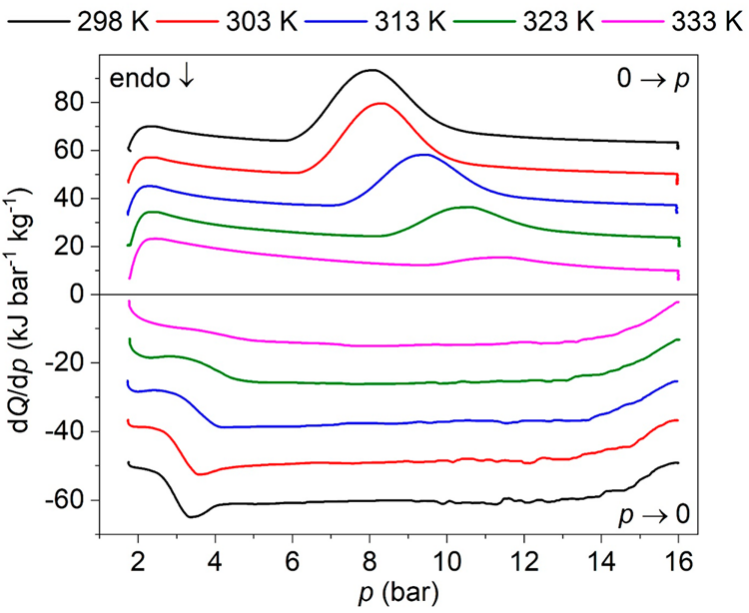
Heat flow d*Q*/d*p* on cycles of applying (0 → *p*) and removing (*p* → 0) CO_2_-pressure at different temperatures (from 298 to 333 K) at the same rate of d*p*/d*t* ∼ 1.6 bar min^–1^. Note: curves have been vertically shifted for facilitating visualization.

However, for practical applications, the compression cycle must be much faster. For that reason, we also carried out continuous isothermal calorimetry experiments on pressurization and depressurization from 1.5 to 16 bar at different temperatures, and at different pressurization/depressurization rates. The obtained results indicate that while temperature highly affects the thermal behavior of the sample (Figure 5), the effect of pressurization rates is almost negligible (Figure S8 of SI). Moreover, they also show that the thermal behavior for a given temperature is reproducible along time under pressurization cycles maintaining quasi-isothermal conditions (see Figure S9 of SI). It should be noted that all experiments have been performed on the same sample, which has maintained its stability and thermal behavior.

Moreover, and as shown in Figures 5 and S8 of SI, in these experiments MIL-53(Al) is seen to exhibit two exothermic peaks on pressurization. At room temperature (*T* ∼ 298 K), the first peak appears at 2.2 bar with an enthalpy change of Δ*H* ∼ 10.9 kJ kg^–1^, whose position is independent from temperature, and it is reversible on depressurization without showing any hysteresis. This peak can be related to the CO_2_ adsorption/desorption in the np-phase, which according to reported isotherms occur at such pressures without any pressurization/depressurization hysteresis.^26^

Meanwhile, a second exothermic peak appears at 8.1 bar upon pressurization at room temperature (*T* ∼ 298 K) with a value of Δ*H* ∼ 81.8 kJ kg^–1^, and it is reversible with a pressure hysteresis of 4.5 bar^–1^. This pressure hysteresis remains constant upon the application of several cycles (see Figure S9 of SI) and is in agreement with the hysteresis observed in the reported CO_2_ BET curves for the breathing transition.^26^ Even more remarkably, this hysteresis is noticeably lower than in hybrid barocaloric materials, where the hysteresis for the pressure-induced structural transition can reach values of up to 380 bar.^42^

Furthermore, this second peak gets shifted toward higher pressures when increasing the temperature up to *T* ∼ 333 K, at the expense of progressively reducing its enthalpy change. In that regard, this peak can be related to the CO_2_ adsorption/desorption arising from the breathing transition between the np-phase and the lp-phase, where the large increase of CO_2_ uptake capacity of the lp-phase provokes this broad and large peak.^26^ Therefore, it can be concluded that the breathing transition is responsible for the large thermal transition occurring at *p* ∼ 8.1 bar. In this latter thermal transition, and as previously discussed, two thermal processes coexist which are opposite in sign, namely the CO_2_ adsorption/desorption and the solid–solid structural transition. In turn, because the resulting peak is exothermic on pressurization, the CO_2_ adsorption should be the dominant contribution. However, it would not happen without the presence of the structural transition, which upon occurring suddenly increases the adsorption capacity of the material.

On the other hand, considering the whole CO_2_ pressurization on to MIL-53(Al) from 1.5 to 16 bar, the total enthalpy change reaches a value of Δ*H* ∼ 92.7 kJ kg^–1^, which is consistent with the total enthalpy change obtained from the point-by-point calorimetric experiments described in the previous section (Δ*H* ∼ 106.8 kJ kg^–1^), even if is slightly lower because here the pressure is applied at a faster rate without giving enough time for complete thermodynamic equilibrium. In any case the fact that most of Δ*H* is achieved, even under short cycle times, is a very favorable result, especially for practical applications.

### 3.3 Analysis of Breathing-Caloric Effects for Potential Refrigeration Applications

The breathing transition of MIL-53(Al) has been previously explored for gas storage^24^ and mechanical energy storage^43,44^ applications, among others. However, this is the first time that the breathing mechanism is experimentally studied for caloric cooling and/or heating effects and, in turn, differs from the well-established magneto-, electro-, elasto-, and barocaloric effects.^7^

Actually, the pressure-induced caloric effects observed here show more resemblance with barocaloric effects than with the others because, in both, a pressurizing fluid applies an isostatic pressure (as external stimulus) to the material, which induces structural transitions and large thermal changes. However, there are also important differences that make it necessary to coin a new term for the caloric effects observed in these breathing transitions: (1) the pressurizing fluid in barocalorics does not chemically interact with them, while the pressurizing gas gets adsorbed in MOF cavities during the breathing transitions, creating new chemical bonds; (2) the pressurizing fluid in barocalorics always favors the lower volume phase, while the pressurizing gas generally favors the larger volume (large-pore) phase in breathing MOFs; (3) the barocaloric effects arise only from the crystalline structural changes, while the caloric effects in breathing MOFs arise from a combination of crystalline structural changes and CO_2_ adsorption.

Therefore, because all these singularities have their origin in the breathing nature of the solid to solid phase transition, we introduce the term “breathing-caloric” to refer to this noticeably different solid-state caloric effect. In this context, to analyze the breathing-caloric effects for potential refrigeration applications, we calculate the caloric effect in terms of isothermal entropy change, which exhibits very large (colossal) values of up to Δ*S* ∼ 311.0 J K^–1^ kg^–1^ at 298 K. This pressure-induced breathing-caloric effect is very superior to most of the pressure-induced barocaloric effects reported in the literature.^5−11,15,16,45^ Even more remarkably, the breathing-caloric effect is fully reversible under the application of very low pressures (*p* = 16 bar,) in contrast to the *p* > 1000 bar required for most barocalorics.^5−11^ Accordingly, the caloric strength of the material, defined as the isothermal entropy change per unit of pressure, Δ*S*/Δ*p* ∼ 19.4 × 10^3^ J K^–1^ kg^–1^ kbar^–1^, is the largest strength reported to date (see below).

**Figure 6:**
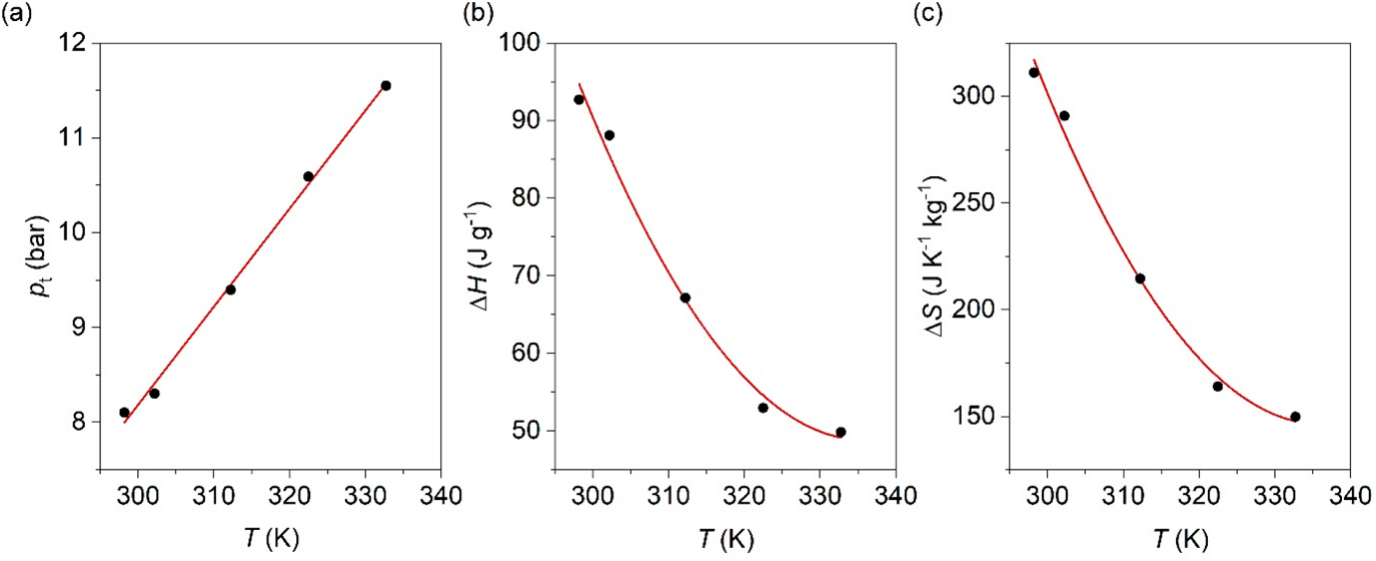
(a) Variation of transition pressure, *p*_t_, of the breathing transition as a function of operating temperature. (b) Variation of Δ*H* as a function of operating temperature. (c) Variation of Δ*S* as a function of operating temperature. Note: data represented from the pressurization curves.

Also, our studies reveal that the operating temperature range (*T*_span_) extends from 333 K down to (at least) 298 K (detection limit of our equipment), as observed in Figures 5 and 6. Interestingly, when decreasing the temperature, the critical pressure for the breathing transition (measured at the maximum of the second peak) decreases from 11.6 bar at 333 K down to 8.1 bar at 298 K, which would imply a lower energy consumption to refrigerate at lower temperatures (see Figure 6a). Therefore, the required pressure is in the range of commercial gas refrigerants for vapor-compression technologies (for instance, *p*(CO_2_) ∼ 64 bar and *p*(R134a) ∼ 6 bar)^46^ and is much lower than in the case of traditional solid barocalorics.^5−11,15,16,45^ In view of these results, MIL-53(Al) shows a temperature span of, at least, 35 K for *p* = 16 bar, which widely exceeds the span of most barocaloric materials.^5−11,15,16,45^

Furthermore, we also observe that Δ*H* and Δ*S* of the whole process increase when decreasing the temperature, from Δ*H* ∼ 49.9 kJ kg^–1^ and Δ*S* ∼ 149.8 J K^–1^ kg^–1^ at 333 K (upper limit of the operating range) up to Δ*H* ∼ 92.7 kJ kg^–1^ Δ*S* ∼ 311.0 J K^–1^ kg^–1^ at 298 K. These results suggest that the caloric refrigeration of this material will be even larger below room temperature (see Figure 6b,c). In that regard, BET isotherms data suggest that MIL-53(Al) can present breathing mechanisms down to 254 K,^26^ which will be the lower limit of the temperature span. Additionally, we also anticipate that it would be possible to further enhance the caloric response by modulating the particle size, shape, and aggregation of the MIL-53(Al) sample, because these factors have been already demonstrated to affect the gas adsorption capacity.^47^

**Figure 7:**
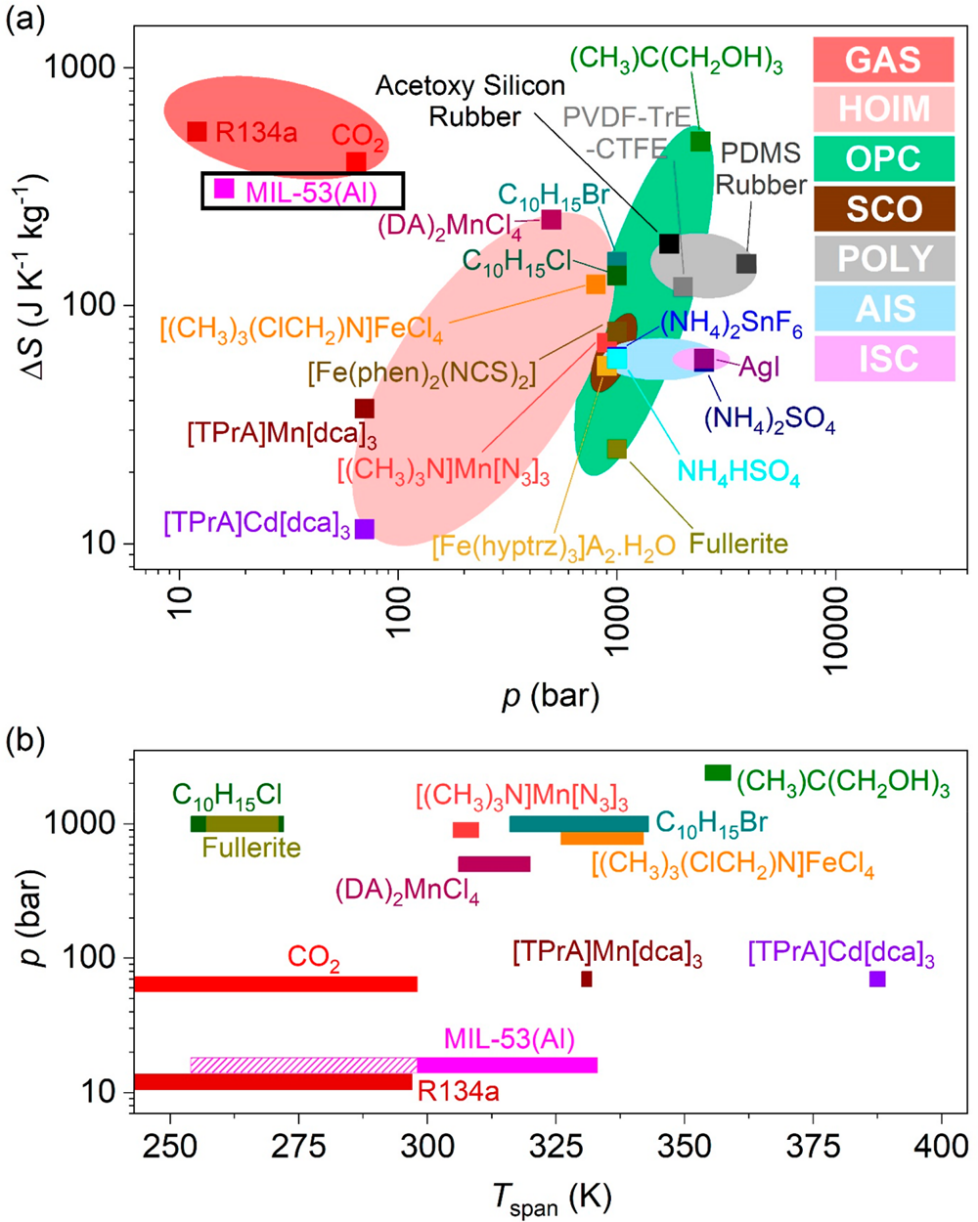
(a) Ashby plot of Δ*S* vs *p* for MIL-53(Al) and different refrigerants.^5−11,15,16,45,46^ (b) Comparison of the operating temperature range (*T*_span_) of the best refrigerants selected from (a). Note: In panel a, GAS = gas refrigerants, HOIMs = hybrid organic–inorganic materials, OPC = organic plastic crystals, SCO = spin crossover materials, POLY = polymers, AIS = ammonium inorganic salts, ISC = ionic superconductors. In panel b, the pink striped area indicates the *T*_span_ below RT for MIL-53(Al) estimated from reported BET isotherms.^26^

In the following paragraphs, we compare the performance of the breathing-caloric effect of MIL-53(Al) reported here with that of selected barocalorics and of very well-known gas refrigerants: the F-gas R134a and stand-alone CO_2_.^46^Figure 7 shows the Ashby plot of isothermal entropy change (Δ*S*) as a function of the required working pressure (*p*) and temperature span (*T*_span_) of these refrigerants. As can be observed, the values of Δ*S* and *p* for MIL-53(Al) are very close to those of the commercial refrigerant gases, while those of the rest of the barocaloric materials are still far away in terms of thermal changes and/or operating pressure. Interestingly, the barocaloric strength (Δ*S*/Δ*p*) of MIL-53(Al) widely exceeds that of the best barocaloric materials and is 200% superior to that of the CO_2_ gas refrigerant, which at 298 K shows a value of Δ*S* ∼ 400 J K^–1^ kg^–1^ and requires *p* ∼ 64 bar to operate (see Figure S10 of SI).^46^ It should be also noted that at temperatures higher than 298 K, the CO_2_ refrigerant needs even higher pressures.

Additionally, *T*_span_ of MIL-53(Al) is also superior to that of the best reported barocalorics^5−11^ and perfectly matches with that of low-temperature CO_2_ systems.^34−37^^46^ Therefore, MIL-53(Al) would be an ideal complementary refrigerant that would operate above 298 K, requiring lower pressure than stand-alone CO_2_. In that way, a combined CO_2_/MIL-53(Al) HVAC system could make use of the cooling capacities of both refrigerants, expanding the operating temperature range (*T*_span_) and reducing the required working pressure.

Overall, the caloric strength of MIL-53(Al) is superior to that of solid barocalorics and CO_2_ gas refrigerant. Furthermore, the operating pressure is similar to that of gas refrigerants and significantly lower than that of barocalorics, which offers a wider temperature span in addition to less energy consumption.

It worth to note that the breathing-caloric effect reported here has similarities to three current refrigeration technologies, namely, vapor-compression, barocaloric, and adsorption cooling. The breathing-caloric effect is similar to vapor-compression and barocaloric refrigeration in that all of them work under pressurization/depressurization cycles that trigger a phase transition. However, these technologies differ in the origin of the generated thermal changes. In the breathing-caloric effect, the main thermal change is originated from an adsorption process, which resembles adsorption cooling technologies. Meanwhile, the thermal change of vapor-compression and barocaloric refrigerants is mainly due a volume change associated with a first-order phase transition. In the breathing-caloric material shown here, the thermal change from the structural transition is opposite in sign to the thermal change from the adsorption mechanism, and this latter change is responsible for the observed caloric effect. However, we anticipate that both lattice and adsorption processes can also be additive, enhancing even further the resulting breathing-caloric effect. For example, the phase diagram (Figure 3) of MIL-53(Al) shows a phase transition from the lp- to the np-phase below ambient pressure and at room temperature. This phase transition involves a decrease of volume and an adsorption process upon compression from very low pressure, and very remarkably, both process should be additive (both exothermic processes). Unfortunately, our DSC equipment is not able to perform measurements under low pressure to confirm this hypothesis.

In any case, and very interestingly, the breathing-caloric effect combines the advantages of both barocaloric and adsorption cooling technologies. For example, the thermal changes are as large as in the case of adsorption technologies, but they are fully reversible upon pressurization/depressurization cycles on a solid-state material, without the need for thermal treatments to desorb the adsorbate and regenerate the adsorbent. This is a clear advantage for designing new refrigeration technologies based on pressurization/depressurization cycles making use of the breathing transitions, not only in MIL-53(Al) but also in any other material with this type of singular phase transitions.

**Figure 8:**
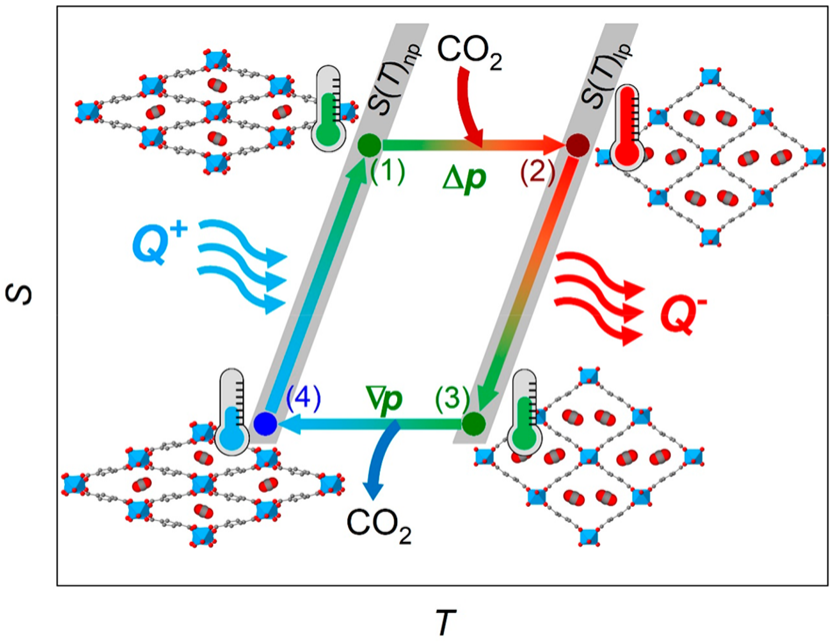
Ideal Brayton cycle that illustrates a possible cooling/heating cycle based on the MIL-53(Al) breathing-caloric effect. The cycle consists of four stages: (1 → 2) adiabatic pressurization of MIL-53, which in turn increases its temperature, (2 → 3) heat release (*Q*^–^) from MIL-53 under isobaric conditions, (3 → 4) adiabatic depressurization of MIL-53, which further decreases its temperature, and (4 → 1) heat from the surroundings (*Q*^+^) is absorbed by MIL-53 under isobaric conditions. Note: the heat released in stage 2 → 3 can be used for heating applications or just discarded as residual heat, while the heat absorbed in stage 4 → 1 is useful to cool a fridge chamber and/or a room in the case of an air conditioning system.

In that regard, for illustration purposes, Figure 8 shows a tentative refrigeration/heating cycle based on the ideal Brayton cycle that is often used to depict caloric effects in solid-state materials.^48^ In the case of MIL-53(Al), this cycle would consist of four stages: In the first stage (1 → 2), there is an adiabatic pressurization of the material using CO_2_, where the MIL-53(Al) adsorbs CO_2_ and increases its temperature. In the second stage (2 → 3), MIL-53(Al) is kept under constant pressure (isobaric conditions) and releases heat (*Q*^–^) to the environment (which can be used for heating applications or just discarded as residual heat). In turn, the MOF decreases its temperature. In a third stage (3 → 4), there is an adiabatic depressurization where MIL-53(Al) desorbs the previously captured CO_2_ molecules and decreases further its temperature. Finally, in the last stage (4 → 1), MIL-53(Al) is kept depressurized (isobaric conditions) and absorbs heat (*Q*^+^) from the surroundings (which could be used to cool a fridge chamber or a room in the case of an air conditioning system).

## 4 Conclusions

Since its discovery, the MIL-53 family has been subjected to intensive study due to its enormous structural flexibility during adsorption–desorption of guest molecules, which induces the well-known breathing transition between two phases: the so-called narrow-pore phase and the large-pore phase. Very interestingly, we have experimentally observed that the breathing transition of MIL-53(Al) material exhibits colossal thermal changes of Δ*S* ∼ 311 J K^–1^ kg^–1^ and Δ*H* ∼ 93 kJ kg^–1^ at room temperature (298 K) and under only 16 bar of CO_2_ gas. Therefore, this material with a breathing transition is very promising for refrigeration applications, owing to its colossal thermal changes, its extremely low operating pressure, and its wide temperature span. Moreover, these findings open up a new path toward eco-friendly refrigeration technologies, coined here as “breathing-caloric”. Very remarkably, this new refrigeration technology combines characteristics of the vapor-compression technology with those of the adsorption and barocaloric technologies. For example, the breathing-caloric technology requires a gas compressor as the current vapor-compression refrigeration, the thermal changes are mainly due to adsorption–desorption processes as the adsorption cooling technology, and it requires the presence of a solid to solid phase transition as the barocaloric technology. In any case, we find that this innovative breathing-caloric refrigeration mechanism offers very large thermal changes (in the range of vapor-compression and adsorption refrigeration) under compression/decompression cycles (typical of vapor-compression and barocaloric refrigeration). At the same time, this mechanism requires significantly low operating pressures (even lower than vapor-compression and barocalorics) and do not require thermal-induced regeneration as typical adsorption materials do. Accordingly, the MIL-53(Al) material exhibits interesting physical–chemical properties very suitable for practical applications in refrigeration. In that regard, we suggest that the practical implementation of breathing-calorics could be straightforward given that they present engineering requirements similar to existing cooling technologies.

Finally, we anticipate that MIL-53(Al) is not an isolated example, but these breathing-caloric effects will appear in more MOFs and other porous materials with breathing transitions. We expect that the results obtained here will encourage the search for new materials with breathing-caloric effects. In addition, this new breathing-calorics technology could be added to the list of emerging technologies that aim to achieve a more efficient and eco-friendly refrigeration.

## Supporting Information Available

Instrumental scheme, DSC, PXRD, FT-IR, TEM, TGA, BET, and materials comparison (PDF)
