# Supplementary material for: Discovery of Colossal Breathing-Caloric Effect under Low Applied Pressure in the Hybrid Organic–Inorganic MIL-53(Al) Material
Source: Chem Mater. 2022 Mar 30;34(7):3323–32. doi: 10.1021/acs.chemmater.2c00137 (PMC9011131; doi:10.1021/acs.chemmater.2c00137)
Supplement: Supplementary file 1 — cm2c00137_si_001.pdf [file cm2c00137_si_001.pdf]

Supporting Information for the manuscript:

Discovery of Colossal Breathing-Caloric Effect under Low Applied Pressure in the Hybrid Organic-Inorganic MIL-53(Al) Material

Javier García-Ben[†,‡], Jorge López-Beceiro[§], Ramon Artiaga[§], Jorge Salgado-Beceiro[†,‡], Ignacio Delgado-Ferreiro[†,‡], Yury V. Kolen'ko[⊥], Socorro Castro-García[†,‡], María Antonia Señarís-Rodríguez[†,‡,*], Manuel Sánchez-Andújar[†,‡,*], and Juan Manuel Bermúdez-García[†,‡,*]

† Quimolmat, Centro de Investigacións Científicas Avanzadas (CICA), Universidade da Coruña, Rúa As Carballeiras, 15071 A Coruña, Spain.

‡ Quimolmat, Departamento de Química, Facultade de Ciencias, Universidade da Coruña, Campus da Zapateira, 15008 A Coruña, Spain.

§ Escuela Politécnica de Ingeniería de Ferrol, Universidade da Coruña, Campus Industrial de Ferrol, 15403 Ferrol, A Coruña, Spain.

⊥ International Iberian Nanotechnology Laboratory (INL), Avenida Mestre José Veiga, 4715-330 Braga, Portugal.

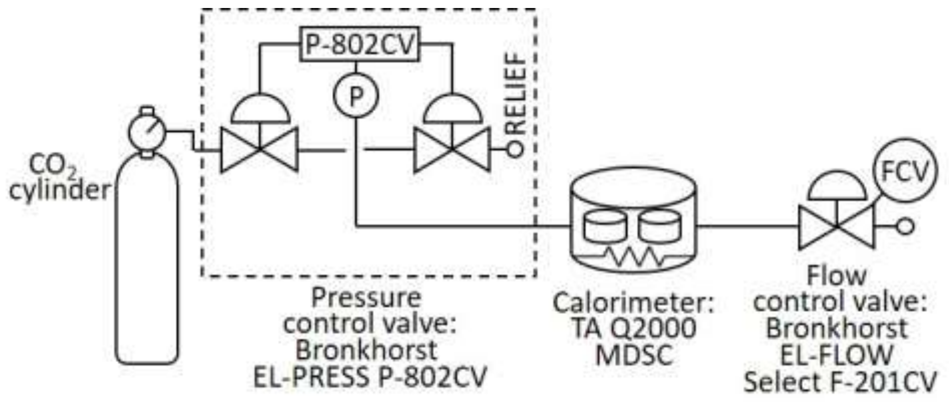

Figure S1. Scheme of the Q2000 MDSC customized with Bronkhorst EL-PRESS P-802CV pressure controller and Bronkhorst EL-FLOW Select F-201CV flow regulator, in the inlet and outlet, respectively.

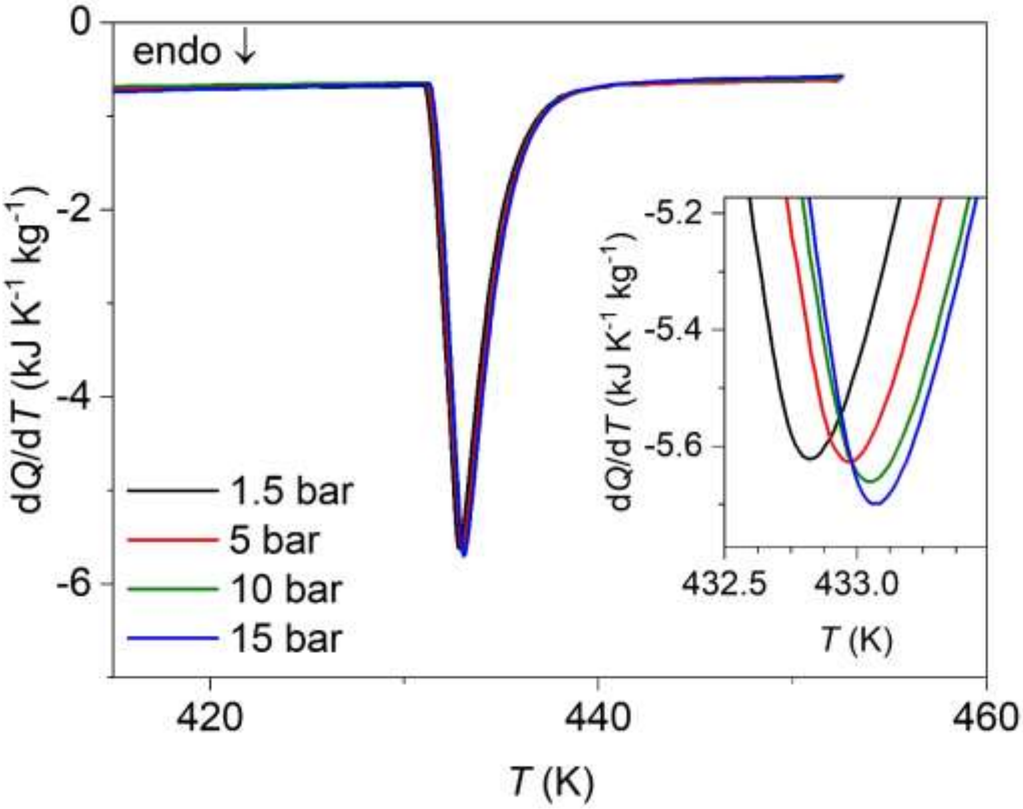

Figure S2. DSC curves of the Indium melting transition at different pressures for pressure calibration purposes following the manufacturer recommendations. Inset: zoom-in of the maximum of the peaks.

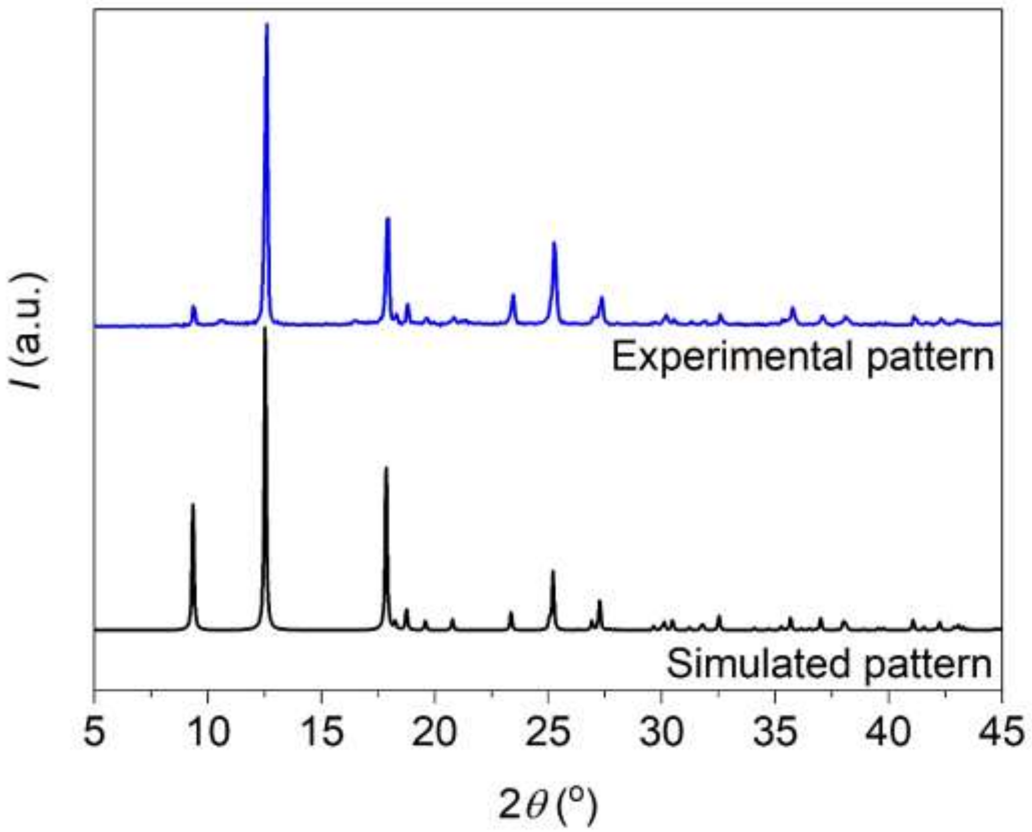

Figure S3. PXRD pattern at ambient conditions of temperature and pressure for the MIL-53(Al) compared with that simulated from reported single crystal XRD data.

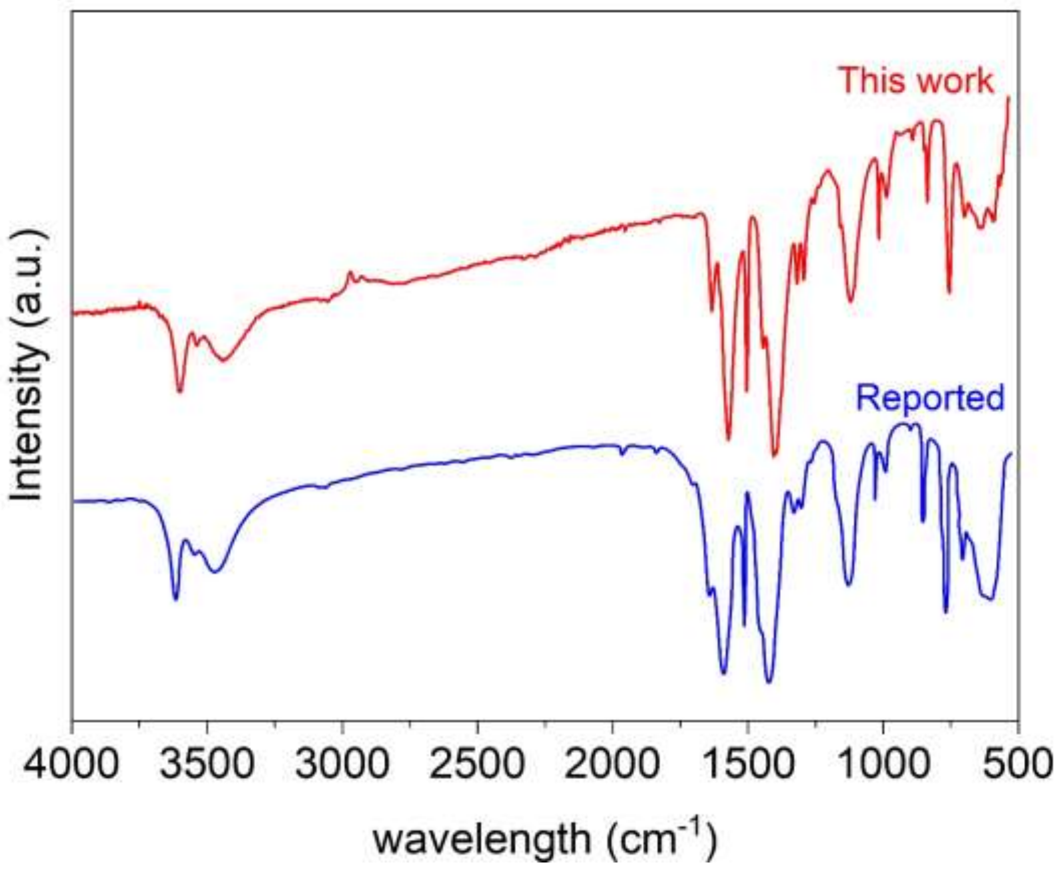

Figure S4. FT-IR spectra of the MIL-53(Al) sample synthesized in this work compared with the spectra reported in the literature under the same conditions.[1]

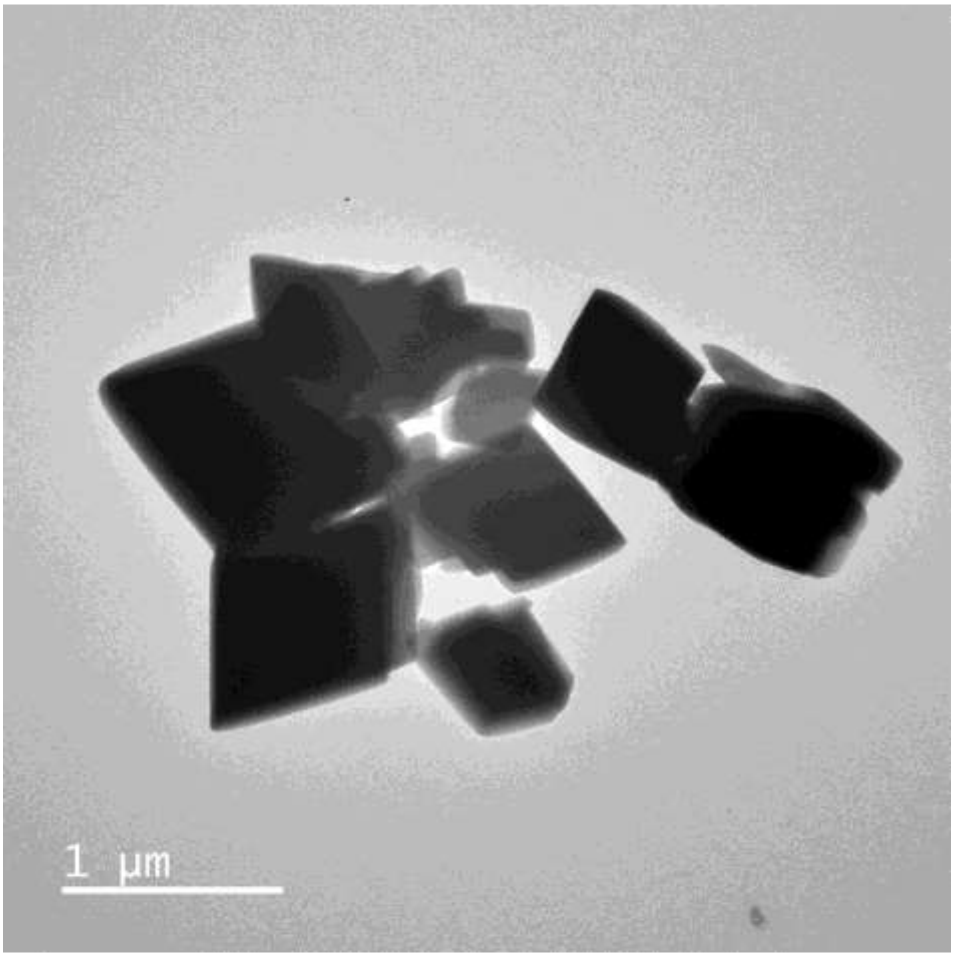

Figure S5. TEM micrograph showing the morphology of the MIL-53(Al) sample.

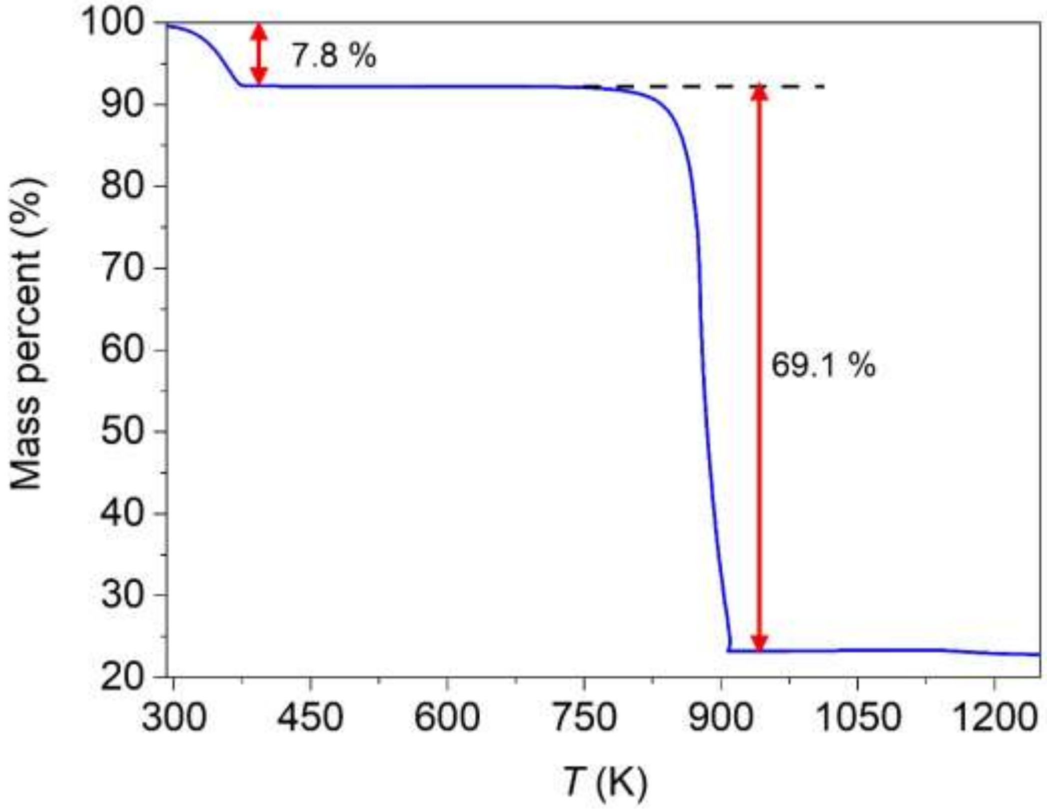

Figure S6. TGA water desorption and decomposition curve for the MIL-53(Al) sample under nitrogen atmosphere.

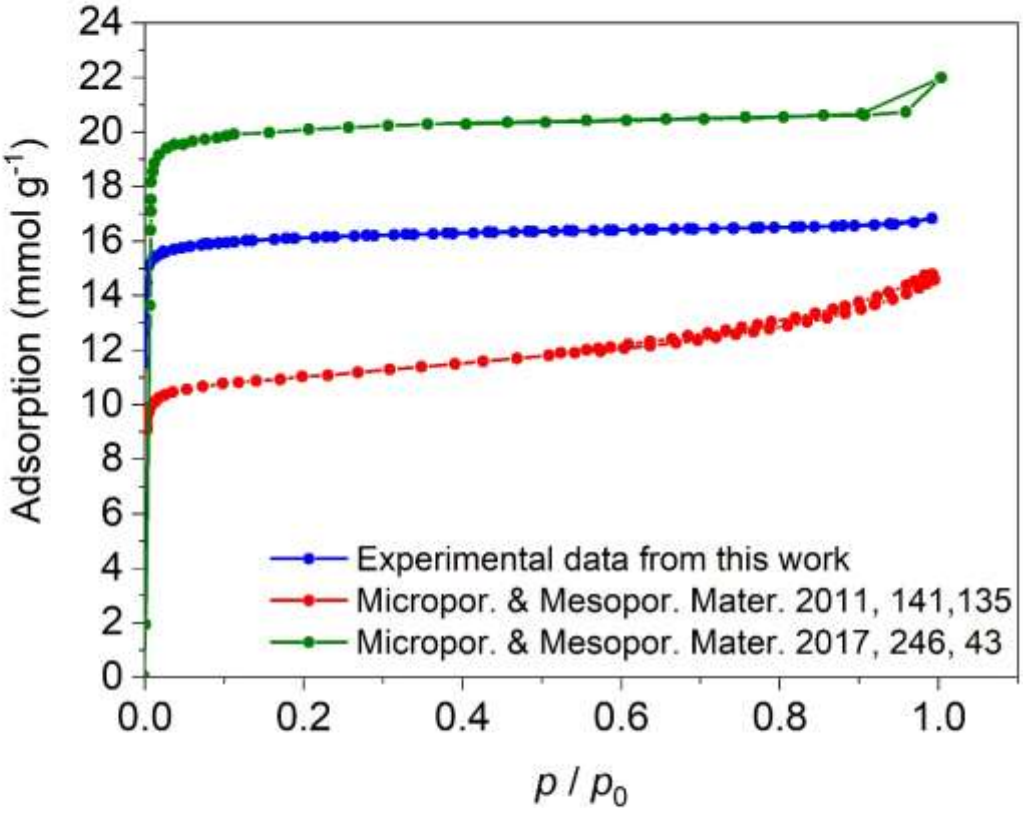

Figure S7. N_2-BET adsorption/desorption isotherms at 77 K.

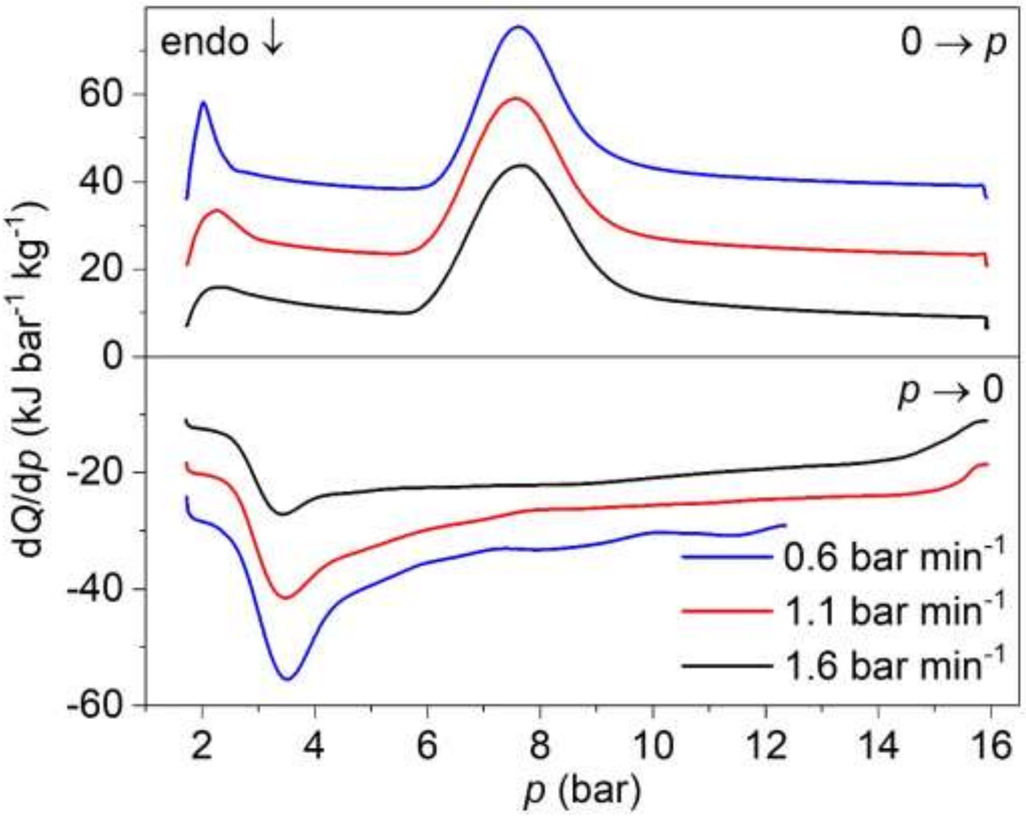

Figure S8. Heat flow d*Q*/d*p* on cycles of applying (0 → *p*) and removing (*p* → 0) CO_2-pressure at different d*p*/d*t* rates (from 0.6 bar min^{-1} to 1.6 bar min^{-1}) at the same temperature, $T = 298$ K. Note: curves have been vertically shifted for facilitating visualization.

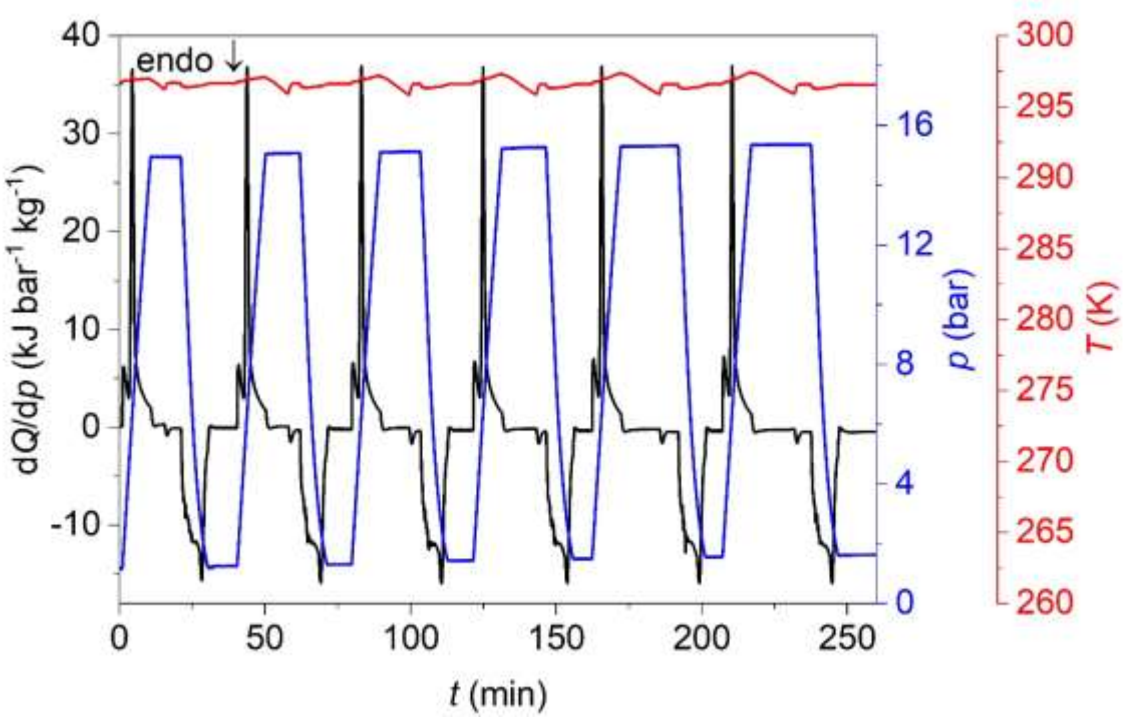

Figure S9. Heat flow on 6 cycles of applying (0 → *p*) and removing (*p* → 0) CO_2-pressure under quasi-isothermal conditions at 298 K and at a rate of d*p*/d*t* ~ 1.6 bar min-^{1}. **Note:** there is a noticeable change of temperature on the isobaric region due to the equipment temperature stabilization when stopping the pressure increase. This change is reflected in a small endothermic peak on the heat flow curve in the isobaric region that falls out (and does not affect to) the pressure-induced breathing-caloric effect region.

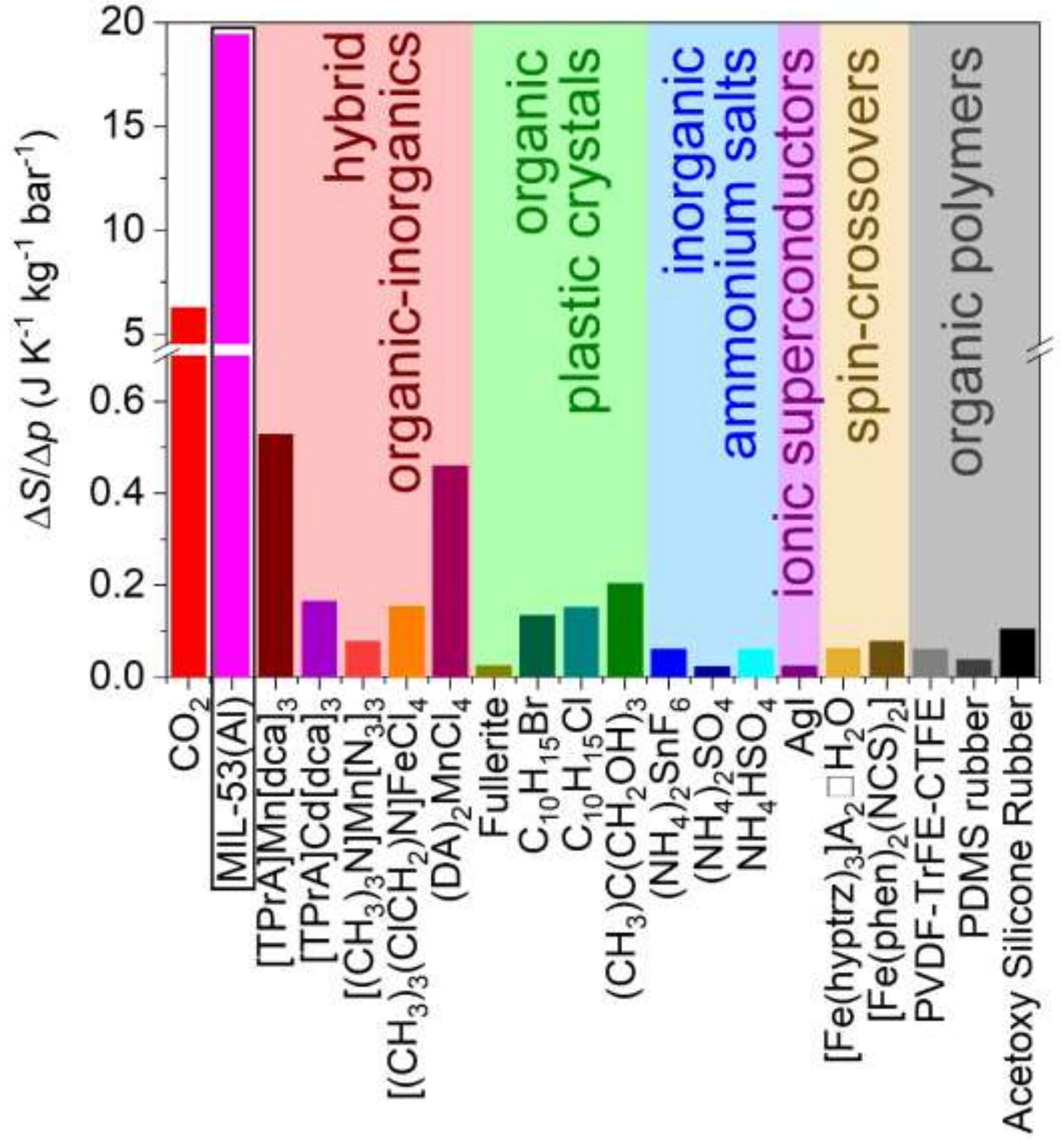

Figure S10. Comparison of caloric strength ($\Delta S/\Delta p$) of the MIL-53(Al) hybrid material with that of the CO_2 gas refrigerant and of the best barocaloric materials reported up-to date.

References:

(1) Loiseau, T.; Serre, C.; Huguenard, C.; Fink, G.; Taulelle, F.; Henry, M.; Bataille, T.; Férey, G. A Rationale for the Large Breathing of the Porous Aluminum Terephthalate (MIL-53) Upon Hydration. *Chem. - A Eur. J.* **2004**, *10* (6), 1373–1382. 10.1002/chem.200305413.
